# Traditional and complementary medicine use among cancer patients in Nepal: a cross-sectional survey

**DOI:** 10.1186/s12906-022-03555-8

**Published:** 2022-03-15

**Authors:** Soo Jeung Choi, Sangita Karki Kunwor, Hyea Bin Im, Jung Hye Hwang, Dain Choi, Dongwoon Han

**Affiliations:** 1 Department of Global Health and Development, Graduate School, Hanyang University, 222 Wangsimni-ro, Seongdong-gu, Seoul, 04763 South Korea; 2 Institute of Health Services Management, Hanyang University, Seoul, South Korea; 3 Department of Obstetrics and Gynecology, Hanyang University College of Medicine, Seoul, South Korea; 4 Department of Preventive Medicine, Hanyang University College of Medicine, Seoul, South Korea

**Keywords:** Cancer, Traditional and complementary medicine, Nepal

## Abstract

**Background:**

Traditional and complementary medicine (T&CM) is commonly used in South Asian countries such as Nepal. There are various causes and contributing factors for patients with cancer to consider using T&CM. However, little is known about the use of T&CM among the cancer population in this region.

**Methods:**

The study followed a cross-sectional design using a structured survey questionnaire. Survey participants were recruited from two National hospitals in Kathmandu, Nepal. The survey instrument comprised 30 questions, including variables on demographics, use of T&CM, and perceived level of disease severity, and cancer treatment. Chi-square test and logistic regression were used for data analysis using SPSS ver. 23.0.

**Results:**

Of 908 participants, 31.6% used one or more modalities of T&CM after a cancer diagnosis. The most commonly used T&CM was Ayurveda (46.5%), followed by yoga (32.4%). About 46% of T&CM users discussed their use with their doctors. The main source of information on T&CM was their family members and relatives (55.7%). Cancer type (head and neck cancer OR: 2.30, CI: 1.23–4.29; abdominal cancer OR: 2.69, CI: 1.47–4.95; lung cancer OR: 5.88, CI: 2.69–12.89), cancer stage (Stage I OR: 1.92¸CI: 1.14–3.25; Stage II OR: 1.76, CI: 1.06–2.94), and the patients’ self-rated disease severity (high perceived severity OR: 1.50, CI: 1.05–2.16) were strong predictors of T&CM use.

**Conclusion:**

This study underlined that despite the widespread use of T&CM among cancer patients in Nepal, most patients obtained information on T&CM from informal sources and did not disclose their use to physicians. To ensure the safe use of T&CM modalities, physicians should integrate questions on T&CM use into routine patient assessments in order to facilitate active communication and improve the quality of care.

## Background

Cancer is still a life-threatening disease for all populations and can pose a greater challenge in resource-limited countries, where cancer diagnosis and treatment are delayed due to the inadequate availability of medical services [1]. Despite global efforts to improve cancer patients’ accessibility to conventional medicine [2, 3], traditional medicine has been generally considered an available and affordable medical resource in less developed countries, such as Nepal [4–6]. Traditional and complementary medicine (T&CM) is an umbrella term that refers to a set of healthcare practices provided outside of the dominant medical system and forms of indigenous medicine such as Ayurveda medicine [7].

The use of T&CM is common in various cultural settings [7], and its use among the cancer patients has increased over the past decades [4, 8, 9]. Since the early 1980s, multiple studies examined patterns or predictors of T&CM utilization among cancer patients [9–11]. The average prevalence of T&CM use in cancer patients has doubled from 25.0% before the 1990s [8] to 51.0% after the 2010s [9]. The increasing trends in T&CM use seem to reflect unmet needs that are commonly identified among cancer patients, associated with their physical and psychological symptoms and quality of life [12]. Given the potential risk of concurrent use with anti-cancer treatment and complementary therapies, identifying the predictors of traditional and complementary medicine (T&CM) use among cancer patients is a significant public health concern [13, 14].

According to the complementary and alternative medicine (CAM) healthcare model [15], individuals perceiving high severity of illness are generally more likely to pursue all kinds of health care services under their beliefs. Patients experiencing more severe symptoms or poor prognoses were more likely to use non-conventional medicine to improve their health [16–18]. However, no previous literature has identified differences in T&CM modalities used by cancer patients depending on the perceived level of disease severity.

Nepal, located in the Hindu Kush Himalayan (HKH) region, has a long history of traditional medicines use, which have been passed down through generations in a community-based belief system [19, 20]. More than half of patients with chronic conditions in Nepal (55.7%) used non-conventional therapies, especially Ayurveda. Furthermore, patients aged 40 or above, with higher education levels, higher family income, residing in cities, and with chronic problems were more likely to rely on self-care approaches [21]. However, no studies have focused on the T&CM use by Nepalese cancer patients. Therefore, this study aims to investigate the prevalence, patterns, and predictors of T&CM used by patients with cancer in Nepal, particularly the differences in the use of T&CM modalities based on the patients’ perceived severity of the disease. This study also explores the doctor-patient communication on T&CM use.

## Methods

### Study design

A cross-sectional study was conducted to assess the T&CM use among cancer patients in Nepal.

### Study setting and participants

The study was conducted at Bhaktapur Cancer Hospital (BCH) and Tribhuvan University Teaching Hospital (TUTH). TUTH is the largest tertiary hospital and provides care in all major specialties to 700 inpatients and more than 2000 outpatients per day. BCH is the only specialized cancer hospital in Kathmandu (the capital city), providing almost 425 outpatient services, including radiation and daycare, emergency care with 110 inpatient services per day. These two hospitals cover almost half of the cancer care services of the nation. Patients in the oncology wards and visiting outpatient clinics during the data collection period were invited to participate in the study. Eligible participants were diagnosed with cancer at the time of the interview, 18 years of age or older, and understood questions in Nepali or English. Exclusion criteria were newly diagnosed cancer patients because cancer diagnosis could not have influenced the patient’s use of T&CM, and the patients who expressed refusal to participate in the survey or had difficulty responding to the survey.

### Study size

The required sample size was calculated using the confidence interval-based sample size determination formula: *n* = Z^2^_α/2_*pq/d^2^, where n is the required sample size, α = 1-CL, Z_**α/2**_ is 1.96, which corresponds to the confidence interval of 95%, d is the margin of error set on 0.035, p is expected proportion based on an average prevalence of T&CM use in South Asian countries (*p* = 0.56) [22–26], and q is the proportion of people not using T&CM (1-p). The calculated sample size was 771, and the total sample size was 930 considering a 20% non-response rate.

### Data collection

The data was collected by two trained nurses through face-to-face interviews using a structured questionnaire from December 2018 and August 2019. All participation was voluntary, and Institutional Review Board (IRB) approved informed consent was obtained before the survey. If the respondents could not read the self- administered questionnaire, a written/signed informed consent were obtained from legal guardian for study participation. A total of 930 cancer patients were invited to participate in the study, and after excluding the incomplete responses, 908 responses were included in the final analysis (a response rate of 97.6%).

### Survey instrument

The questionnaire was first developed in the English language based on previous studies investigating T&CM use among cancer patients [23, 27–30], and the content validity of the questionnaire was tested by four experts (two researchers who previously conducted similar studies in Korea and two research advisors from TUTH Research Center, Nepal). A Nepalese researcher then translated the questionnaire into the Nepali language, and it was converted back into English to verify its accuracy. Lastly, the pilot test was performed with 20 participants to improve its clarity and evaluate the reliability of the modified questionnaire.

The final version of the questionnaire consisted of four sections with 36 items, including multiple-choice and open-ended questions. The first section included 11 questions on the medical characteristics of participants, such as cancer types, stage of cancer, type of conventional treatment, and health behaviors obtained from the medical records. The second section contained Likert-type scales to assess the patient’s current health status, including general health condition (1 = poor, 5 = excellent), and the level of disease severity and perceived discomfort due to conventional cancer treatment (1 = not at all severe, 10 = extremely severe). The third section includes eleven questions regarding T&CM use, which contain the patterns of T&CM use, the reason for T&CM use, the perceived effectiveness of T&CM, side effects of T&CM, the sources of information about T&CM, and the disclosure of T&CM use to physicians. T&CM modalities were categorized into three different groups based on their type (i.e., natural products such as herbal products, honey, vitamins, and minerals; mind and body practices such as yoga, meditation, massage, prayers, and spiritual process; and the complementary approaches such as Ayurveda and traditional healers). Furthermore, the last section consists of ten questions on the socio-economic characteristics of respondents (gender, age, educational level, employment status, perceived-economic status, area of residence, religion, ethnicity, marital status, housing type, and family structure).

### Statistical analysis

The collected data were summarized using Statistical Package for Social Sciences (SPSS) version 23.0. Pearson’s Chi-square test was used to identify associations between T&CM use and variables; gender, age groups (≤49, 50–59, ≥60), levels of education (no formal education, basic education and above), place of residence (city, municipality, village), employment (housewife, employed), economic status (enough, barely enough, inadequate), types of cancer (hematologic cancer, urogenital cancer, women’s cancer (ovary/breast), head and neck cancer, abdominal cancer, and lung cancer), and received conventional treatments (surgery, radiotherapy, chemotherapy, and others). Items measured on a 10-point scale were categorized into two groups based on the average response value: self-perceived disease severity (average response: 6.11; low-perceived disease severity: 1–6; high-perceived disease severity: 7–10) and level of discomfort due to conventional cancer treatments (average response: 2.79; low-level of discomfort: 1–3; high-level of discomfort: 4–10). Lastly, multivariate logistic regression analysis was conducted to determine potential predictors of T&CM use among cancer patients. The significant factors from the previous chi-square test (age, education, residential area, employment status, type of cancer, cancer stage, type of conventional cancer treatment (CCT), self-rated disease severity, perceived discomfort in CCT were included in the regression analysis. In addition, only the data of 784 respondents with specific cancer type (i.e., non-metastatic cancer) was included in the regression analysis because cancer type, which is one of the significant factors from the chi-square analysis, only includes the data of patients with localized cancer.

### Ethical clearance

The study was performed in compliance with the Declaration of Helsinki. The Institutional Review Board on Human Subjects Research and Ethics Committees at Hanyang University (HYI-18-164-2) and Institutional Review Committee at Tribhuvan University (437 (6–11) E2 /075/76) approved the study.

## Results

### Sociodemographic and clinical characteristics of study participants

The sociodemographic and clinical characteristics of the respondents are shown in Table 1. The mean age of the respondents was 53.7 ± 15.6 years (range 18–92), and the ratio of males and females was similar. The majority were respondents without a spouse (69.3%), employed (56.4%), without any formal education (78.3%), and lived outside the city (82.5%). The most frequent types of cancer were abdominal cancer (22.8%), followed by head and neck cancer (20.3%), and hematologic cancer (18.3%).

**Table 1:** Socio-demographic and clinical characteristics of participants

Variables	Total ***N*** = 908 (%)	T&CM users ***N*** = 287 (31.6)	Non-users ***N*** = 621 (68.4)	***P***-value
Gender				
Male	450 (49.6)	151 (52.6)	299 (48.1)	0.211
Female	458 (50.4)	136 (47.4)	322 (51.9)	
**Age (Mean, SD)**	53.7 (15.6)	55.5 (13.4)	52.8 (16.4)	< 0.001
≤ 49	327 (36.0)	83 (28.9)	244 (39.3)	0.005
50 ~ 59	220 (24.2)	84 (29.3)	136 (21.9)	
≥ 60	361 (39.8)	120 (41.8)	241 (38.8)	
Spouse				
Yes	274 (30.2)	89 (31.0)	185 (29.8)	0.710
No	634 (69.3)	198 (69.0)	436 (70.2)	
**Education level**				
No formal education	711 (78.3)	207 (72.1)	504 (81.2)	0.002
Basic education and above	197 (21.7)	80 (27.9)	117 (18.8)	
Residing area				
City	159 (17.5)	25 (8.7)	134 (21.6)	0.001
Municipality	391 (43.1)	124 (43.2)	267 (43.0)	
Village	358 (39.4)	138 (48.1)	220 (35.4)	
Employment				
House wife	396 (43.6)	83 (28.9)	313 (50.4)	< 0.001
Employed	512 (56.4)	204 (71.1)	308 (49.6)	
Economic Status				
Enough	205 (22.6)	77 (26.8)	128 (20.6)	0.060
Barely enough	571 (62.9)	176 (61.3)	395 (63.6)	
Inadequate	132 (14.5)	34 (11.8)	98 (15.8)	
Cancer progression				
Metastasis	124(13.7)	45(15.7)	79(12.7)	0.227
Localized	784(86.3)	242(84.3)	542(87.3)	
Type of cancer (localized cancer only) ^a^				
Hematologic cancer	166 (21.2)	144 (86.7)	22 (13.3)	< 0.001
Urogenital cancer	72 (9.2)	56 (77.8)	16 (22.2)	
Women’s cancer (ovary/breast)	102 (13.0)	77 (75.5)	25 (24.5)	
Head and neck cancer	184 (23.5)	117 (63.6)	67 (36.4)	
Abdominal cancer	207 (26.4)	128 (61.8)	79 (38.2)	
Lung cancer	53 (6.8)	20 (37.7)	33 (62.3)	
Cancer stage				
Unclassified	121 (13.3)	48 (16.7)	73 (11.8)	< 0.001
Stage I	202 (22.2)	92 (32.1)	110 (17.7)	
Stage II	73 (8.0)	30 (10.5)	43 (6.9)	
Stage III	77 (8.5)	27 (9.4)	50 (8.1)	
Stage IV	435 (47.8)	90 (31.4)	345 (55.6)	
Type of conventional medicines				
Surgery				
Yes	365 (40.2)	100 (34.8)	265 (42.7)	0.025
No	543 (59.8)	187 (65.2)	356 (57.3)	
Radiotherapy				
Yes	283 (31.2)	106 (36.9)	177 (28.5)	0.011
No	625 (68.8)	181 (63.1)	444 (71.5)	
Chemotherapy				
Yes	384 (42.3)	126 (43.9)	258 (41.5)	0.504
No	524 (57.7)	161 (56.1)	363 (58.5)	
Others				
Yes	121 (13.3)	41 (14.3)	80 (12.9)	0.563
No	787 (86.7)	246 (85.7)	541 (87.1)	
Self-rated disease severity ^b^				
Mean (SD)	6.11(2.08)	6.40 (2.11)	5.97 (2.06)	0.003
Low	475 (52.3)	121 (42.2)	354 (57.0)	< 0.001
High	433 (47.7)	166 (57.8)	267 (43.0)	
**Level of perceived discomfort due to CCT** ^**c**^				
Mean (SD)	2.79 (1.04)	2.93 (0.82)	2.73 (1.13)	0.009
Low	399 (43.9)	86 (30.0)	313 (50.4)	< 0.001
High	509 (56.1)	201 (70.0)	308 (49.6)	

*T&CM* Traditional & Complementary medicine, *CCT* conventional cancer treatment

^a^ The sum of this variable is 784 because it only includes localized cancers

^b^ Self-rated disease severity was categorized two groups: 1 ~ 6: Low, 7 ~ 10 High

^c^ level of discomfort due to CCT was categorized two groups: 1 ~ 3: Low, 4 ~ 10 High

### Use of T&CM

**Fig. 1:**
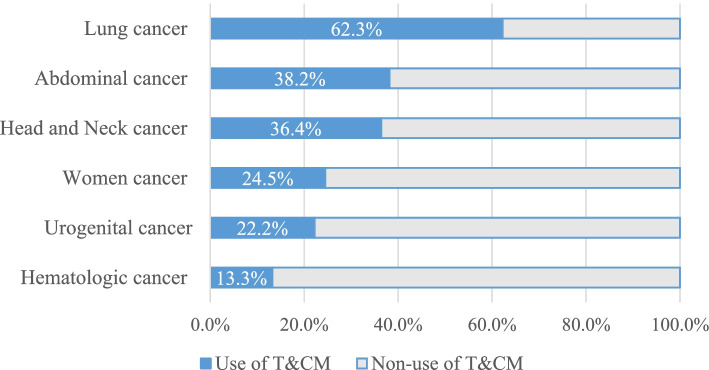
Frequency of T&CM use by cancer diagnostic group

Overall, 31.6% (*n* = 287) of participants used at least one type of T&CM (Table 1). Significant differences between T&CM users and non-users were found in age (*p* = 0.005), education level (*p* = 0.002), employment status (*p* <  0.001), and residential area (*p* = 0.001). In addition, we found that the type of cancer (*p* <  0.001), cancer stage (*p* <  0.001), type of treatment such as surgery (*p* = 0.025) and radiotherapy (*p* = 0.001) were associated with T&CM use. The respondents rated their self-perceived severity and the discomfort level due to conventional cancer treatment as 6.11 ± 2.08 and 2.79 ± 1.04, respectively, which were significantly higher among T&CM users (*p* <  0.001). As Fig. 1 presents, there were significant differences in T&CM use among the different cancer diagnostic groups of the respondents (*p* <  0.001). The highest prevalence of T&CM use was observed among lung cancer patients (62.3%), followed by abdominal cancer patients (38.2%), and the patients with head and neck cancer (36.4%).

### T&CM modalities

The most common types of CAM, reportedly used by respondents, were Ayurveda (46.5%), yoga (32.4%), herbal products (30.9%), meditation (28.9%), and honey (23.4%). Table 2 shows the T&CM modalities used by respondents based on the self-rating of disease severity. Among natural products, herbal product use was the highest (30.9%), followed by honey (23.4%). Consumption of honey (*p* <  0.001) and ginger (*p* <  0.001) was significantly greater in the group with a higher rating of disease severity. For mind and body practices, yoga, meditation, and praying was most common among respondents (32.4, 28.9, 12.5%, respectively), and it was also found that yoga (*p* <  0.001) and meditation (*p* <  0.001) were significantly higher in the group with the greater perception of disease severity. Among other complementary health approaches, consumption of Ayurveda was the most prevalent (46.5%), followed by traditional healers (18.4%).

**Table 2:** T&CM Modalities used by cancer patients based on their perceived level of disease severity

T&CM Modalities	N (%)^a^ *n* = 287	High perceived severity^b^ *n* = 166 (%)	Low perceived severity^b^ *n* = 121 (%)	*P*-value
**Natural Products (*N*** **= 269)**				
Herbal products	79 (30.9)	42 (26.8)	37 (37.4)	0.073
Honey	60 (23.4)	53 (33.8)	7 (7.1)	< 0.001
Ginger	58 (22.7)	49 (31.2)	9 (9.1)	< 0.001
Tulsi	56 (21.9)	44 (28.0)	12 (12.1)	0.003
Cow-urine	9 (3.5)	7 (4.5)	2 (2.0)	0.302
Vitamins/Minerals	7 (2.7)	3 (1.9)	4 (4.0)	0.309
**Mind and Body Practices (*n*** **= 202)**				
Yoga	83 (32.4)	72 (45.9)	11 (11.1)	< 0.001
Meditation	74 (28.9)	67 (42.7)	7 (7.1)	< 0.001
Pray	32 (12.5)	21 (13.4)	11 (11.1)	0.594
Massage	4 (1.6)	3 (1.9)	1 (1.0)	0.571
Spiritual Process	9 (3.5)	5 (3.2)	4 (4.0)	0.717
**Other Complementary Health Approaches (*N*** **= 166)**				
Ayurveda ^c^	119 (46.5)	68 (43.3)	51 (51.5)	0.200
Traditional Healers	47 (18.4)	28 (17.8)	19 (19.2)	0.785

^a^ Columns do not add up to 100% due to the selection of multiple answers

^b^ Self-rated disease severity was categorized two groups: Low (1 ~ 6), High (7 ~ 10)

^c^ Ayurveda: only provided by Ayurveda practitioner

### Source of information on T&CM

As presented in Table 3, the main source of information on T&CM as reported by the cancer patients was their family members and relatives (55.7%), followed by friends (27.2%), and billboard advertisements/magazines/TV/radio (7.1%). Only, 6.3% of respondents obtained T&CM information from T&CM providers.

**Table 3:** Patterns of T&CM use after cancer diagnosis

Variables	N	%
**Sources of Information about T&CM (*N*** **= 287)**		
Family/relatives	160	55.7
Friends	78	27.2
Hooding board /Magazines/TV/Radio	21	7.3
T&CM providers	18	6.3
Internet	6	2.1
Health workers	2	0.7
Others	2	0.7
**Reasons for using T&CM (*N*** **= 287)**^**a**^		
Desire to do everything possible to fight the disease	157	54.7
Relief from symptoms	141	49.1
To improve general condition	138	48.1
To improve immune function	114	39.7
To support emotional well-being	106	36.9
Belief in advantages of T&CM	87	30.3
To fight the disease directly	53	18.5
Family tradition/culture	44	15.3
Dissatisfaction with CCT	39	13.6
To desire to take a control of treatment	27	9.4
Requested by physician	9	3.1
Other	15	5.2
**Reasons for not using TCM (*N*** **= 621)**		
Not sufficiently informed about its efficacy	214	34.5
Satisfaction with CM	190	30.6
Unproven benefit as cancer treatment	112	18.0
Not recommended by doctor	85	13.7
Dissatisfaction with TCM	15	2.4
More expensive than conventional treatment	4	0.6
Other	1	0.2
**Disclosure of T&CM use to doctor (*N*** **= 287)**		
Yes	132	46.1
No	155	53.9
**Reason for non-disclosure (*N*** **= 155)**		
Doctor never asked	53	34.2
Doctor would discourage T&CM use	35	22.6
Doctor won’t understand	22	14.2
It is not important to disclosure	20	12.9
None of the doctor business	12	7.7
Others	13	8.4

*CCT* conventional cancer treatment

^a^ Columns do not add up to 100% due to the selection of multiple answers

### Reasons for T&CM use and non-disclosure of T&CM use to physicians

The most frequently stated reasons for T&CM use were due to patients’ desire to do everything possible to fight the disease (54.7%), to relieve symptoms (49.1%), to improve the general condition (48.1%), and to improve immune function (39.7%). Among non-users, the primary reason was insufficient information on the efficacy of T&CM (34.5%), followed by satisfaction with conventional medicine (30.6%). Of the 287 participants that used T&CM, 46.1% disclosed their physician of T&CM use. The most reported reason for non-disclosure of T&CM use was the doctors not asking about the use (51.7%), followed by the fear of doctors discouraging T&CM use (22.6%), and the concern over the doctor not understanding their use (14.2%) (Table 3).

### Predictors of T&CM use

The results from multivariate logistic regression analysis are shown in Table 4, and it revealed that patients with lung cancer were 5.88 times more likely to use T&CM than patients with hematologic cancer (CI: 2.69–12.89, *p* <  0.001). In addition, suffering from abdominal cancer (OR: 2.69, CI: 1.47–4.95) or head and neck cancer (OR: 2.30, CI: 1.23–4.29), early-stages of cancer (Stage I OR: 1.92¸CI: 1.14–3.25; Stage II OR: 1.76, CI: 1.06–2.94), and having higher perceived disease severity (OR: 1.50, CI: 1.05–2.16) were positively associated with the utilization of T&CM.

**Table 4:** Factors associated with T&CM use

Variables	OR	95% CI	***P***-value
**Age**			
≤ 49	1	Ref	
50–59	1.459	0.940–2.263	0.092
≥ 60	1.083	0.724–1.622	0.697
**Educational level**			
No formal education	1	Ref	
Basic education and above	1.438	0.955–2.163	0.082
**Residing area**			
City	1	Ref	
Municipality	1.676	0.977–2.875	0.061
Village	1.660	0.944–2.919	0.079
**Employment**			
House wife	1	Ref	
Employed	1.161	0.747–1.805	0.506
**Type of cancer (localized cancer only)**			
Hematologic cancer	1	Ref	
Urogenital cancer	1.639	0.743–3.614	0.221
Women’s cancer (ovary/breast)	1.468	0.720–2.994	0.291
Head and neck cancer	2.299	1.233–4.286	0.009
Abdominal cancer	2.693	1.466–4.946	0.001
Lung cancer	5.884	2.687–12.887	< 0.001
**Cancer stage**			
Unclassified	1	Ref	
Stage I	1.924	1.138–3.253	0.015
Stage II	1.761	1.056–2.938	0.030
Stage III	1.604	0.831–3.099	0.159
Stage IV	1.336	0.722–2.475	0.356
**Type of CCT**			
**Surgery**			
Yes	1	Ref	
No	0.988	0.650–1.503	0.956
**Radiotherapy**			
Yes	1	Ref	
No	0.975	0.643–1.478	0.905
**Self-rated disease severity**			
Low	1	Ref	
High	1.504	1.047–2.159	0.027
**Perceived discomfort in CCT**			
Low	1	Ref	
High	1.177	0.753–1.839	0.474

*CCT* conventional cancer treatment

## Discussion

To our knowledge, this is the first study to explore the prevalence and patterns of T&CM use among cancer patients in Nepal. The overall prevalence of T&CM use among Nepali cancer patients was 31.6%, and among the cancer groups, the prevalence of T&CM ranged from 13.3% (hematologic cancer) to 62.3% (lung cancer). The finding was in accordance with a previous study [4], a systematic review of T&CM use in cancer patients in low-income and lower-middle-income countries.

The use of T&CM was associated with various factors such as education, residence, cancer stage, and perceived disease severity, which are in line with the previous studies such as a higher level of education [27, 29, 31, 32], residing outside the urban [6], the early stage of cancer [18], and the higher perceived disease severity [18]. Interestingly, the prevalence rates of T&CM use were different based on cancer types and stages. Despite suggestions from the literature that T&CM use is significantly higher in the group with advanced diseases and recurrent diseases [33], some studies showed that the tumor stage is not associated with the use of alternative therapies [28, 34]. Moreover, the present study showed that patients with advanced cancer stage were less likely to use T&CM. These results suggest that cancer stages affecting the use of T&CM are varied. These variations between studies may be explained by various causes such as the attitude of oncologists, cultural and religious beliefs, the cost of conventional treatment, and the questionnaire used to collect the data [35].

It was also interesting to see the prevalence of T&CM use in Nepal, where a wide range of modalities such as Ayurveda, spiritual healers (Dhami-Jhankri), and self-treatment with medicinal plants, have been officially established as a part of traditional medical systems [6]. The results showed that the prevalence of T&CM use is similar to a previous study in Germany (29.0%) [36], Turkey (33.8%) [28], South Korea (37.5%) [37] and India (38.7%) [22]. However, the prevalence of T&CM use in Nepal was lower than those reported in Mongolia (47.9%) [27], and Italy (48.9%) [29]. The potential reasons for variations between the results are the heterogeneity of study designs, such as differences in definitions of T&CM [11, 13, 29], or in sampling strategies that cause selection bias [11, 38]. Another possible explanation may be that cancer patients from resource-limited countries, such as Nepal [39], may not attend cancer treatment facilities [4].

As a part of T&CM, Ayurveda is commonly used in South Asian countries, such as Nepal and India. This is not unexpected, as many patients seek traditional medicine in Nepal and India [7]. Congruent with the results reported in India [25], nearly half of cancer patients using T&CM have taken Ayurveda. In addition, our results showed that the patients with a higher perceived level of disease severity are more likely to practice yoga/meditation and consume honey, ginger and tulsi than the group with lower perceived severity. An interesting key finding was that patients who perceived cancer as serious are more likely to use T&CM. Therefore, we further analyzed how respondents used different T&CM modalities based on their perceived level of disease severity. Unlike general preference, cancer patients with higher self-rated severity were more likely to use yoga, meditation, honey, ginger, and tulsi than the group with lower self-rated severity. The high popularity of natural products can be attributed to the patients’ belief that those modalities are safe [22, 40, 41]. However, natural products are not completely natural and safe in all cases [42]. For example, ginger, which is one of the most widely cultivated herbs in Nepal, is known to be effective for managing chemotherapy-induced nausea [43]; yet, when co-dosed with aprepitant, it is found to aggravate nausea [44, 45]. Therefore, caution should be taken when it is taken concurrently with conventional medicine. Furthermore, the popularity of natural honey consumption among cancer patients suggests that although the health benefits of honey on cancer are well documented [46], further research is required to examine its potential role in alleviating cancer-related symptoms.

Our findings are consistent with previous studies conducted in developing countries [9, 47], which showed that the most commonly reported reasons for cancer patients’ use of T&CM are due to its curative and holistic effects, beliefs in T&CM, and dissatisfaction with conventional medicine [27, 41]. Thus, patients’ perception of T&CM as a cure for cancer [24, 44] is associated with a delay in seeking appropriate cancer treatment, especially in less developed countries with limited health resources [24, 48]. Moreover, a previous study reported the risk of interactions between T&CM and conventional medicine, which may have serious clinical consequences [36]. Therefore, clinicians should be aware of and make patients aware of the potential interactions in developing countries.

Considering the prevalence of T&CM use among cancer patients [13, 27] and T&CM’s potential interactions with conventional medicines [49], physician-patient communication on T&CM use is important. However, T&CM use is rarely discussed with conventional health care providers, and the communication on T&CM use is most likely to be initiated by the patient [50]. Moreover, including this study, patients’ disclosure rates to their physicians were still considerably low, ranging from 40 to 50% [13]. The reason for not disclosing their use to physicians primarily depends on how the patient perceives their physician’s attitude, such as oncologists’ indifference (lack of inquiry) and opposition (fear of physician’s disapproval) towards T&CM [13, 51, 52]. Additionally, the physicians may feel inadequacy in their skills and knowledge to counsel patients on T&CM use [53]. Thus, the physicians should consider patients’ cluster differences in disclosure, their attitude towards T&CM use, and gain cancer patients’ confidence in delivering healthcare services [54].

Similar results have been reported in India [25], where the common source of T&CM information was family and friends. On the other hand, the Western study showed that the media is the primary source of information followed by family and friends [29], whereas a low proportion of cancer patients obtain information on T&CM use from healthcare professionals [27, 55]. Moreover, oncology patients are more likely to continue using T&CM in the hospital setting even though they are uncertain about the efficacy and effectiveness of the non-conventional medicine use [56]. This can be a significant concern for the patients whose use was neither supported by the scientific evidence nor gained the approval from the healthcare professionals [9]. Furthermore, healthcare professionals’ lack of knowledge on T&CM may result in them responding negatively to patients’ use and queries regarding T&CM [57, 58].

As for the information regarding T&CM, the primary sources were informal sources like family, friends, or other patients. The proportion of nonprofessional group in our findings (82.9%) was higher than Indonesia (70.9%) [55], India (67.9%) [59], as well as Australia (77.0%) [60], the United States (60.0%) [61]. While medical personnel accounts for a significant proportion as the source of information in the United States (17.8%) and Australia (39.0%), but in Asia, it is meager like our result (0.7%) or Indonesia (7.3%) [55]. It can be explained by reflecting socio-cultural traditions, which the tradition of “family” in South-East Asia is strong and economic influences [62].

Oncology patients are likely to continue to use T&CM, notwithstanding the lack of scientific knowledge of T&CM or the disapproval from their health professionals [9]. Even though many cancer patients use T&CM, they might not be convinced that their choice is appropriate [56]. Therefore, considering potential issues related to the interaction between T&CM and conventional treatment, it is important to encourage two-way communication between health professionals and patients.

This study shows value in light of the following limitations and strengths. First, as we only evaluated patients who attended hospital settings, which would exclude patients who make do without conventional cancer treatment are not well represented in this study, it might reduce generalizability. Second, in terms of methodology, it is also possible that the use of the face-to-face interview could have influenced the participant’s response, or there may be a recall bias for experience (e.g., discrepancies between what the patient used and remembered as T&CM). Despite these limitations, our findings call for further studies in other Hindu Kush Himalayan countries.

## Conclusions

The finding of this study highlights that T&CM is widely used among cancer patients in Nepal. Despite the wide popularity of T&CM use among cancer patients, a lack of consultation and disclosure of T&CM use to healthcare providers suggests a need to raise awareness on the importance of open communication between the patients and the healthcare professionals.

Most commonly used T&CM among Nepali cancer patients include Ayurveda medicine, yoga, and herb/herbal products. The present study also demonstrates the association between T&CM use and the self-rated severity of cancer conditions. Significant differences were found in the preferred T&CM modalities between the group with a higher rating of the perceived disease severity and the group with a lower rating of the perceived severity. Moreover, T&CM users’ primary sources of information are their family members, relatives, and friends, and nearly half of the patients do not inform their physicians of their T&CM use. Given the danger of potential interactions between cancer and T&CM therapies, as well as the limited amount of research in this area that has been conducted to date, it is necessary to systematically evaluate the effectiveness, education, and safety of the use of T&CM. Furthermore, health care providers should stay up to date on the evidence of T&CM use in cancer and investigate the possible effects of T&CM on patients’ prognoses.

## Data Availability

The datasets generated and/or analysed during the current study are available from the corresponding author upon reasonable request.

## Abbreviations

T&CM: Traditional and complementary medicine
CCT: Conventional cancer treatment
IRB: Institutional Review Board
HKH: Hindu Kush Himalayan
